# Immunomodulatory Effects of Cylindrospermopsin in Human T Cells and Monocytes

**DOI:** 10.3390/toxins15040301

**Published:** 2023-04-20

**Authors:** Antonio Casas-Rodríguez, Óscar Cebadero-Dominguez, María Puerto, Ana María Cameán, Angeles Jos

**Affiliations:** Area of Toxicology, Faculty of Pharmacy, University of Seville, 41012 Seville, Spain; acasasr@us.es (A.C.-R.); ocebadero@us.es (Ó.C.-D.); camean@us.es (A.M.C.); angelesjos@us.es (A.J.)

**Keywords:** cylindrospermopsin, immunotoxicity, THP-1 cells, Jurkat cells, in vitro toxicity

## Abstract

Cylindrospermopsin (CYN) is a cyanotoxin with an increasing occurrence, and therefore it is important to elucidate its toxicity profile. CYN has been classified as a cytotoxin, although the scientific literature has already revealed that it affects a wide range of organs and systems. However, research on its potential immunotoxicity is still limited. Thus, this study aimed to evaluate the impact of CYN on two human cell lines representative of the immune system: THP-1 (monocytes) and Jurkat (lymphocytes). CYN reduced cell viability, leading to mean effective concentrations (EC50 24 h) of 6.00 ± 1.04 µM and 5.20 ± 1.20 µM for THP-1 and Jurkat cells, respectively, and induced cell death mainly by apoptosis in both experimental models. Moreover, CYN decreased the differentiation of monocytes to macrophages after 48 h of exposure. In addition, an up-regulation of the mRNA expression of different cytokines, such as interleukin (IL) 2, IL-8, tumor necrosis factor-alpha (TNF-α) and interferon-gamma (INF-γ), was also observed mainly after 24 h exposure in both cell lines. However, only an increase in TNF-α in THP-1 supernatants was observed by ELISA. Overall, these results suggest the immunomodulatory activity of CYN in vitro. Therefore, further research is required to evaluate the impact of CYN on the human immune system.

## 1. Introduction

In recent years, climate and nutrient changes have contributed to global eutrophication and the proliferation of harmful algal blooms (HABs) [1]. Among these HABs, cyanobacteria are a group of photosynthetic prokaryotes that are present in different geographical areas around the world, living in a broad range of environments, from freshwater and marine ecosystems to terrestrial ecosystems [2]. In addition, Cyanobacteria produce secondary bioactive metabolites known as cyanotoxins. These compounds can reach humans in different ways, mainly orally through contaminated food and water, although inhalation and dermal exposure during recreational activities are also common [3].

Cyanotoxins have toxic effects on human and animal health, which can cause acute and chronic diseases [4]. Among these toxins, microcystins (MCs) are the most frequently studied and commonly detected worldwide [5]. Furthermore, cylindrospermopsin (CYN) is gaining importance due to its increasing occurrence and expansion, in line with CYN-producing species [6]. CYN is a stable alkaloid formed by a tricyclic guanidine moiety combined with a hydroxymethyluracil group needed for its toxicity. This cyanotoxin is produced mainly by *Cylindrospermopsis raciborskii* and *Chrysosporum ovalisporum* and, due to its zwitterionic nature, is a highly water-soluble compound [7]. CYN can induce cytotoxicity [7], genotoxicity [6,8], neurotoxicity [9], endocrine disruption [10] and developmental toxicity [11]. Some of the key effects are on the liver and kidneys. Concerning the mechanisms of action, CYN is well known for its inhibition of protein and glutathione synthesis, and the induction of oxidative stress, and cytochrome P450 seems to mediate its toxicity [12,13]. Furthermore, CYN has been classified as a potential immunotoxin [14,15]. However, reports dealing with CYN’s immunotoxic properties are scarce compared to MC-LR, as reviewed by Diez-Quijada et al. [16], and the underlying mechanisms are still not fully elucidated.

In the last few years, the in vitro immunomodulatory effects of CYN have been studied on human lymphocytes [14,15,17,18,19], human neutrophils [17,20], fish leucocytes [21], fish phagocytic cells [22] and the murine macrophage, RAW264.7 cell line [23,24]. Considering that the immune system is specialized in the defense against pathogens and is composed of a collection of cells and tissues distributed throughout the body [25], research on additional human in vitro models is necessary to complete the immunotoxicological evaluation of CYN. In that sense, THP-1 is a monocytic cell line, in this case isolated from a leukemic young boy. Monocytes are components of the innate immune system and among the primary cells involved in inflammation [26]. The activation of this cell type causes the release of large amounts of pro-inflammatory cytokines and chemokines. It was suggested that THP-1 cells were useful in examining inflammatory responses mediated by drugs [27].

Another important point to justify using THP-1 cells is that they are a model of primary macrophages. Therefore, these cells can be differentiated from monocytes into macrophages using a differentiation agent such as the phorbol-12-myristate-13-acetate (PMA). Accordingly, the effect of CYN on monocytes and the involvement of CYN in the differentiation process could be evaluated [27]. However, to the best of our knowledge, no studies have been conducted to examine the effects of CYN on this cell line. Actually, the experts in the immune system point out the importance of investigating different cell types simultaneously and carrying out comparative studies to better understand cell-type-specific effects [28]. In that sense, another cell line that can be used to investigate the effects of CYN on the immune system is the Jurkat cell line. These cells have similar parameters to human T-lymphocytes and may be a representative model of the adaptive response [29]. Moreover, this cell line is widely used in in vitro T cell signal transduction, cytokines, and receptor expression studies. It can provide reference and guidance for the treatment of various infectious diseases, and research on their pathogenesis [30]. However, no previous studies with CYN have been performed in this cell line.

The innate and adaptive immune systems consist of effector cells producing cytokines (interleukins, chemokines, interferons, and other mediators) that facilitate communication among both systems [31]. Some of these cytokines are the interleukins 2, 6 and 8 (IL-2, IL-6 and IL-8), the tumor necrosis factor α (TNF-α) or the type II interferon (INF-γ). IL-2 is mainly produced by T cells and is involved in their activation and proliferation. IL-6 is a pro-inflammatory interleukin produced by T cells, macrophages, or endothelial cells. This interleukin is implicated in lymphoid differentiation and IgG production. IL-8 is one of the most widely studied chemokines and a critical inflammatory mediator. TNF-α participates in phagocyte cell activation (pro-inflammatory signaling), and IFN-γ is an anti-viral that increases the macrophage activation, the neutrophil and monocyte function and the expression of the major histocompatibility complex 1 and 2 (MHC-1, MHC-2) on T cells [32]. Unfortunately, knowledge about the effects of CYN on these cytokines is scarce. Regarding immune system models, Sieroslawka et al. [22] reported that CYN could influence the gene expression of proinflammatory cytokines, such as IL-1β and TNF-α, in phagocytes from the common carp. Furthermore, CYN increased the production of TNF-α in RAW264.7 cells, but the toxin did not affect IL-6 expression [24].

On the other hand, it is known that CYN can induce concentration and time-dependent cell morphological changes, the inhibition of cell proliferation, and the reduction of cell viability [7]. However, the effects of CYN on cell death mechanisms (apoptosis/necrosis) in immune cells are minimal. In that sense, Poniedzialek et al. [14,15] reported that CYN could produce apoptosis and necrosis in lymphocytes after 6 h (h) of exposure, although at 72 h, only necrotic cells were found. Moreover, Sieroslawska and Rymuszka [33] confirmed that CYN induced apoptosis and necrosis in a leucocyte cell line. Apoptosis was also found in RAW264.7 cells treated with CYN [23].

Considering that in 2015, a strategic US EPA document declared that there is a lack of data on CYN on immunological endpoints [34] and that reports of immunotoxic effects induced by CYN are still not enough, as revealed by the review performed by Diez-Quijada et al. [16], further toxicological research on this topic is required. Thus, this work aimed to investigate for the first time the potential immunotoxic effects in vitro of CYN on Jurkat and THP-1 cells, two representative cell lines of the human immune system. In that sense, cytotoxicity was evaluated in both cell lines. The influence of CYN on monocyte differentiation was assayed in co-exposure with PMA. Flow cytometry was carried out in both cell lines to determine the apoptosis and necrosis after CYN exposure. The gene expression of different cytokines (IL-2, IL-6, IL-8, TNF-α, IFN-γ) was investigated by real-time polymerase chain reaction (RT-qPCR), and the levels of these cytokines in the cell culture medium was measured by ELISA.

## 2. Results

### 2.1. Cell Viability Determination and Influence of CYN on the Differentiation of THP-1 Monocytes into Macrophages

**Figure 1:**
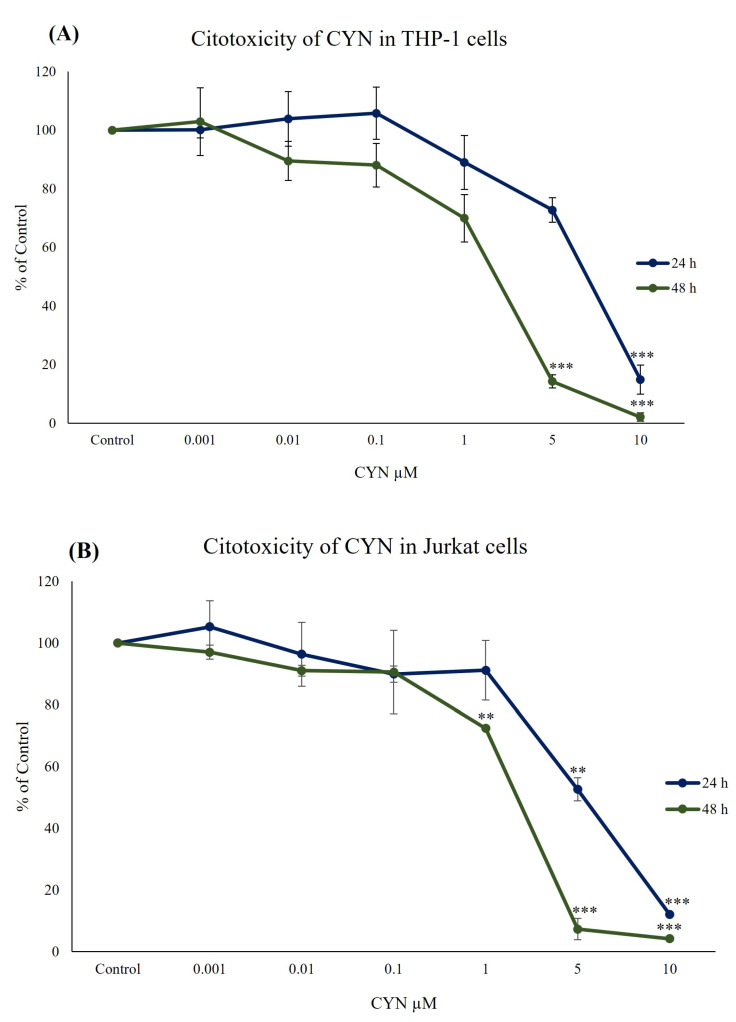
Reduction of tetrazolium salt on (**A**) THP-1 and (**B**) Jurkat cells after 24 h and 48 h of exposure to 0–10 µM CYN. Values are expressed as mean ± SD. All experiments were performed at least three times and at least in triplicate per concentration. The significant levels observed are ** *p* < 0.01 and *** *p* < 0.001 compared to the control group.

The results for cytotoxicity assays for THP-1 and Jurkat cells are shown in Figure 1. In both cells, the exposure to CYN caused a viability decrease from 1 to 10 µM after 24 and 48 h of exposure. However, significant differences were found in THP-1 cells from 5 and 1 µM for 24 and 48 h, compared with the control group. EC50 values were 6.00 ± 1.04 µM for 24 h and 2.96 ± 0.67 µM for 48 h. Jurkat cells were slightly more sensitive to CYN than THP-1, with EC50 values of 5.20 ± 1.20 and 2.32 ± 0.16 µM for 24 and 48 h, respectively.

**Figure 2:**
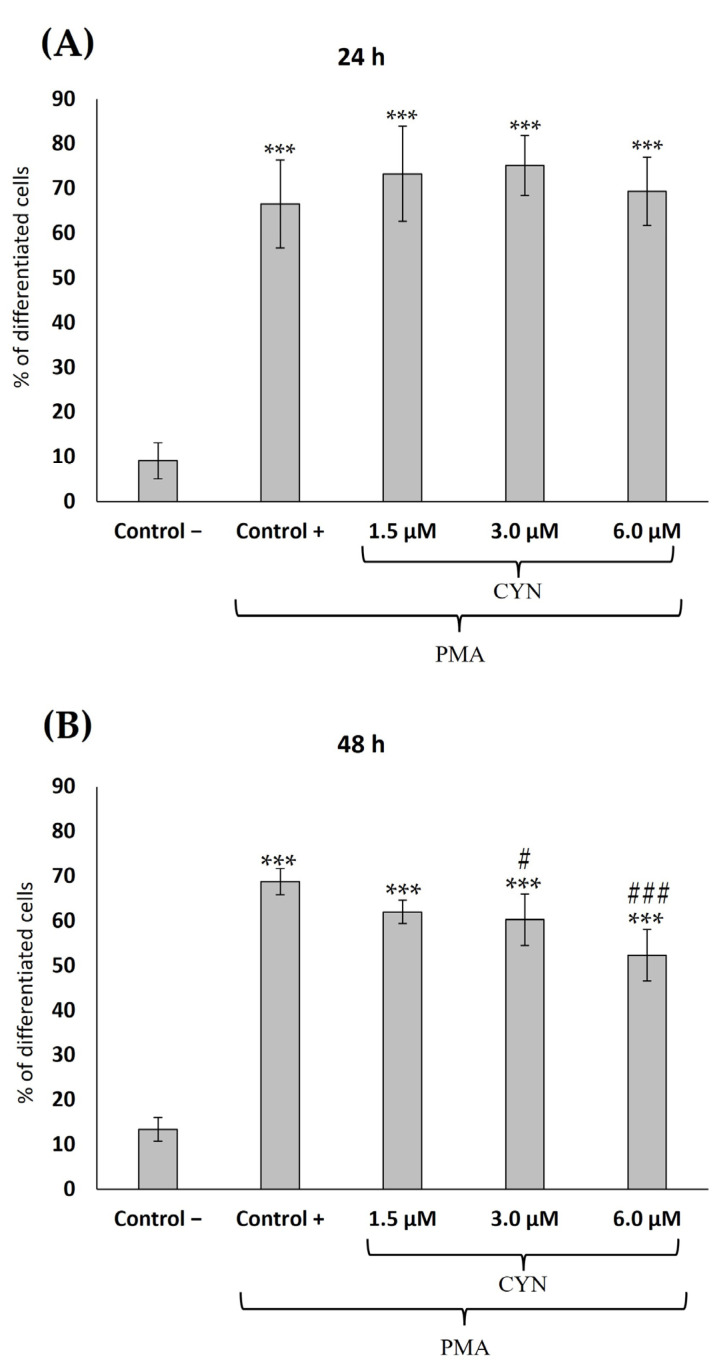
Influence of CYN on the differentiation of THP-1 monocytes to macrophages after (**A**) 24 and (**B**) 48 h of exposure to 1.5 (EC50/4), 3.0 (EC50/2), and 6.0 (EC50) µM CYN. All values are expressed as mean ± SD. The significant levels observed are *** *p* < 0.001 compared to the undifferentiated negative control, # *p* < 0.05, and ### *p* < 0.001 compared to the control group treated with PMA.

Concerning the differentiation assay, THP-1 cells were treated with PMA to differentiate monocytes into macrophages. The influence of different concentrations of CYN 1.5, 3.0, and 6.0 µM (EC50/4, EC50/2, and EC50, respectively) on that process is shown in Figure 2.

For the control without PMA, we obtained 90.85 ± 4.03% and 86.61 ± 2.65% of undifferentiated cells, whereas only 9.12 ± 4.03% and 13.38 ± 2.65% of differentiated cells were observed for 24 and 48 h, respectively. A high increase, as expected, in differentiated cells was found in control cells treated with PMA; significant differences compared to control cells without PMA were found at 24 and 48 h. The values of differentiated cells in the control treated with PMA were 66.54 ± 9.82% and 68.77 ± 2.94% at 24 and 48 h, respectively. For the different CYN concentrations, at 24 h no significant differences in comparison to control cells treated with PMA were observed, with a range between 24.86 and 30.61% of undifferentiated cells. At 48 h, a concentration-dependent effect was found with an increment in the percentage of undifferentiated cells as CYN concentration increased. The percentages of undifferentiated cells (48 h) were 37.98 ± 2.58% (EC50/4), 39.72 ± 5.76% (EC50/2) and 47.63 ± 5.77% (EC50). Significant differences in comparison to control cells treated with and without PMA were found in the 3 and 6 µM CYN-treated groups at 48 h.

### 2.2. Influence of CYN on Cell Death Mechanisms (Apoptosis/Necrosis) by Flow Cytometry

**Figure 3:**
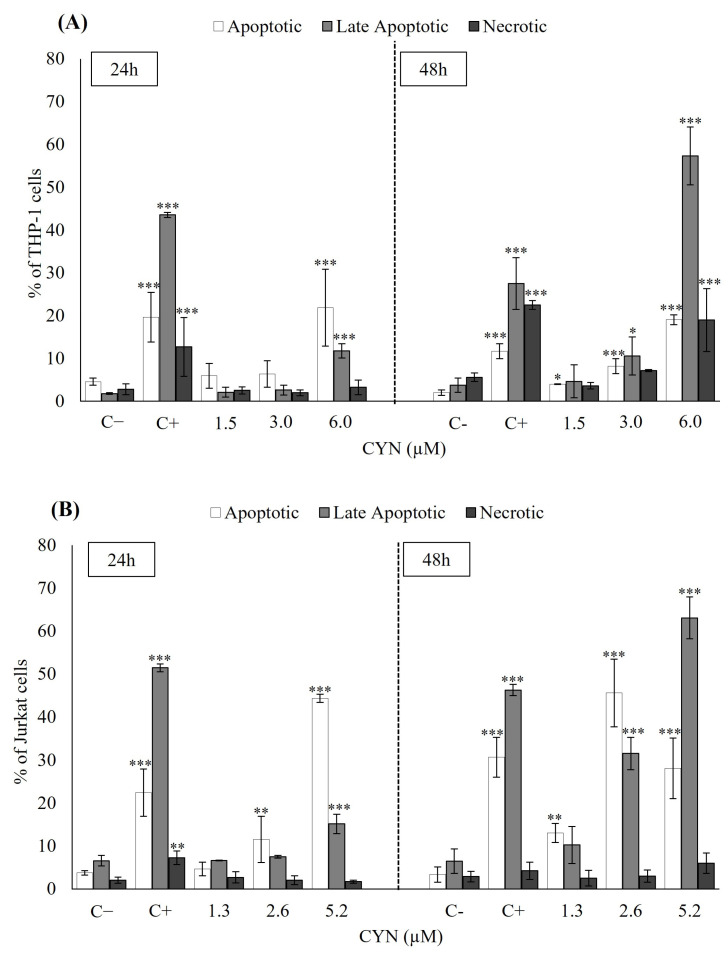
Percentage of apoptosis, late apoptosis, and necrosis in (**A**) THP-1 and (**B**) Jurkat cells after exposure to different concentrations of CYN at 24 and 48 h detected by flow cytometry. Medium-treated cells were used as negative control, and camptothecin (1.5 µM) as positive control. Data are expressed as mean ± SD of three replicates. The significant levels observed are * *p* < 0.05, ** *p* < 0.01 and *** *p* < 0.001 compared to the negative control group.

The effects of CYN on the apoptosis/necrosis of THP-1 and Jurkat cell lines were studied. The different populations of cells were registered with the markers Annexin V-FITC conjugated (apoptosis) and Propidium Iodide (necrosis). The results are shown in Figure 3A for THP-1 and Figure 3B for Jurkat cells. The concentrations studied were the EC50, EC50/2 and EC50/4 of CYN for Jurkat (5.2, 2.6 and 1.3 µM) and THP-1 (6.0, 3.0 and 1.5 µM) cells. Regarding THP-1, more than 90% of cell viability was found in the negative control group. In the positive control group, around 60–70% of cells were apoptotic, late apoptotic, or necrotic after 24 and 48 h. At 24 h, no significant differences were detected at low concentrations of CYN (1.5 and 3.0 µM). Still, at the highest concentration (6.0 µM), a significant increase in comparison to the negative control was found in apoptosis (21.92 ± 8.98%) and late apoptosis (11.81 ± 1.65%). In the case of CYN exposure for 48 h, the lowest concentration only significantly increased the apoptotic cells. At 3.0 and 6.0 µM, significant changes appeared in apoptosis and late apoptosis stages, predominantly late apoptosis after 6.0 µM of exposure. Moreover, at this concentration, the cells may also die via necrosis (18.99 ± 7.32%) (Figure 3A).

Similarly, around 90% of the cell viability was found in the negative control in Jurkat cells. In contrast, in the positive control, approximately 80% of cells were in apoptosis, late apoptosis or necrosis after 24 and 48 h. At 24 h, no significant differences were detected at the lowest concentration of CYN (1.3 µM). However, a concentration-dependent increment in the percentage of cells in apoptosis and late apoptosis was found, and apoptosis was the predominant mechanism with values of 13.51 ± 2.55% and 44.35 ± 0.91% for 2.6 and 5.2 µM CYN, respectively. Significant changes were also observed in the percentage of late apoptotic cells at the highest CYN concentration. At 48 h, in the 1.3 µM exposure group, significant differences were found only in apoptosis (13.01 ± 2.21% of cells). At 2.6 µM, significant differences compared to the negative control were found in apoptosis and the late apoptosis stage, with apoptosis the primary death mechanism with values of 45.59 ± 7.82% of cells. Finally, at the highest concentration assayed, increments in apoptosis (28.09 ± 7.03% of cells) and late apoptosis (63.07 ± 4.86%) were significant. CYN did not induce necrosis at any concentrations or times assayed in Jurkat cells.

### 2.3. Effects of CYN on the Different Cytokines’ Genetic Expression (mRNA Levels) by RT-qPCR

**Figure 4:**
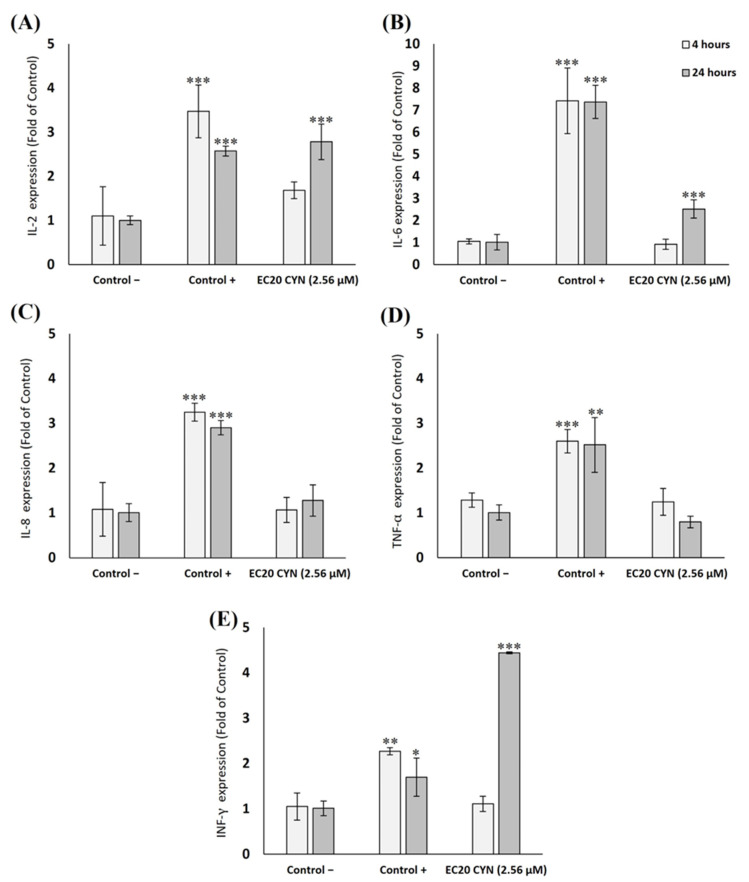
Gene expression of (**A**) IL-2, (**B**) IL-6, (**C**) IL-8, (**D**) TNF-α, and (**E**) IFN-γ expressed as multiples of control in THP-1 cells after 4 and 24 h exposure to CYN effective concentration 20 (EC20) (2.56 µM). Values are expressed as mean ± SD. The significant levels observed are * *p* < 0.05, ** *p* < 0.01 and *** *p* < 0.001 compared to the negative control group. C+: LPS 10 ng/mL.

**Figure 5:**
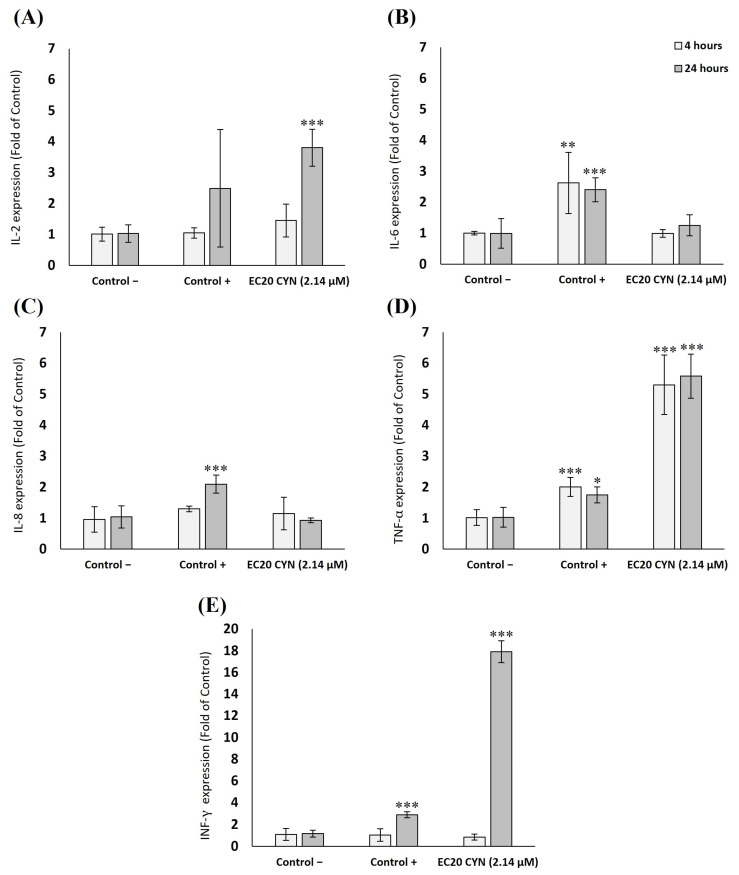
Gene expression of (**A**) IL-2, (**B**) IL-6, (**C**) IL-8, (**D**) TNF-α, and (**E**) INF-γ expressed as fold of control in Jurkat cells after 4 and 24 h exposure to CYN effective concentration 20 (EC20) (2.14 µM). Values are expressed as mean ± SD. The significant levels observed are * *p* < 0.05, ** *p* < 0.01 and *** *p* < 0.001 compared to the negative control group. C+: LPS 10 ng/mL.

Results from the relative gene expression of the studied genes in THP-1 and Jurkat cell lines are represented in Figure 4 and Figure 5, respectively. Regarding THP-1 cells, only a slight pro-inflammatory response after 4 h was found in IL-2 (1.5-fold increase), but no significant differences were found compared to the negative control group. No alterations were observed in the gene expression of IL-6, IL-8, TNF-α and INF-γ after 4 h of exposure to CYN. After 24 h, no changes were found for IL-8 and TNF-α. However, results showed a significant upregulation in the expression of IL-2 (2.9-fold increase), IL-6 (2.5-fold increase) and INF-γ (4.4-fold increase), and significant differences in comparison to the negative control group were found.

In the case of Jurkat cells (Figure 5), similar results were obtained after 4 h of exposure to CYN, where most of the genes showed values close to the negative control (IL-2, IL-6, IL-8 and INF-γ). Compared to the negative control group, only a significant upregulation was observed in the expression of TNF-α (5.2-fold). However, after 24 h of CYN exposure, significant upregulation was found in IL-2 (3.7-fold), TNF-α (5.4-fold) and INF-γ (15.4-fold). At the same time, IL-6 and IL-8 expressions remained similar to the negative control.

### 2.4. Effects of CYN on Cytokines Levels by ELISA

Cytokines were detected by ELISA in supernatants of THP-1 and Jurkat cells after 4 and 24 h of exposure to EC20 of CYN. Cells treated with 10 ng/mL LPS were used as a positive control. For THP-1 cells (Table 1), only TNF-α was detected after the exposure to CYN for 4 h and 24 h and a significant increase was only observed after 4 h for TNF-α. For the other ones, the values were out of range (lower than the detection limit of the test). Similarly, in the case of Jurkat cells, all cytokines measured were found out of range.

## 3. Discussion

The immunological response comprises many different cell types; therefore, several immune cell types and cell lines must be included for immunotoxicity testing to cover a wide range of immunotoxin modes of action [35]. Toxicological in vitro investigations have demonstrated the potential adverse effects of CYN in eukaryotic cells. Nevertheless, only two studies have been carried out in a model of mammalian innate immunity originating from cancerous cells, the RAW264.7 cell line [23,24]. In contrast, the immunotoxic effects of CYN have been studied on human peripheral blood lymphocytes and neutrophils isolated from healthy donors [14,15,17,18,19,20]. Still, donor viability and high individual variation can make analyses and interpretations of these results more complex. In our case, two classical human cell lines, THP-1 and Jurkat, have been used to study and compare CYN’s immunomodulatory effects, increasing the understanding of biochemical mechanisms.

Cytotoxicity assays are usually the first step when carrying out toxicity evaluations in vitro. Our results showed that CYN triggered a viability decrease in both cell lines. Although no data about the effects of CYN on the viability of THP-1 and Jurkat cells have been found in the scientific literature, Moosova et al. [24] performed three cytotoxicity assays (MTS, neutral red uptake and the determination of LDH release) in RAW264.7 macrophages exposed to the toxin (0.5–1 µM). They did not observe significant differences in cell viability after 24 h of exposure. Nevertheless, in the same cell line, Takser et al. [23] found that 10 µM CYN caused a total reduction in viability after 24, 48 and 72 h of toxin exposure. These decreases agree with the data found in the present work for THP-1 and Jurkat cells at similar cyanotoxin concentrations. Furthermore, Zegura et al. [19] carried out other viability assays in human peripheral blood lymphocytes (HPBLs). They found that the exposure to 0.5 µg/mL (1.2 µM) of CYN for 4 or 24 h did not significantly affect the cell viability. However, 1 µg/mL (2.4 µM) of CYN reduced the viability of these lymphocytes in a time-dependent manner [15]. Finally, two studies in lymphocytes and neutrophils did not find significant effects on the number of viable cells after 1 h of exposure to 1 µg/mL of CYN [17,20]. Considering all these results, CYN seems to exert different effects on the viability of different immune cells depending on the concentration and time exposure.

On the other hand, it is interesting to point out that although, in general, the cytotoxic effects found in both cell lines were quite similar, cell viability was shown to be slightly more affected in Jurkat than in THP-1 cells, as the EC50 values obtained were lower after 24 and 48 h of exposure. Moreover, the evaluation of the cell death mechanisms (apoptosis/necrosis) after exposure to CYN were also studied. Although THP-1 and Jurkat cells were used to determine apoptotic and necrotic effects of other toxins [36,37], there are no data about the influence of CYN on the cell death mechanisms of these cell lines. In the present work, we found different responses between THP-1 and Jurkat cells in these death processes (i.e., different apoptosis levels at similar concentrations of the toxin or a more pronounced increase in late apoptosis in Jurkat than in THP-1). Still, the most important difference was that THP-1 cells appeared to die via necrosis after the toxin exposure. In contrast, CYN did not induce necrosis in Jurkat cells at any of the concentrations tested. In this regard, other authors demonstrated that CYN induced apoptosis and necrosis in lymphocytes (1 µg/mL (2.4 µM)) and leucocytes (1 and 10 µg/mL) after 24 and 48 h of exposure, respectively [14,15,33]. Nevertheless, Poniedzialek et al. [20] found that 1 µg/mL of CYN had no significant effect on the percentage of apoptotic and necrotic neutrophils compared to the negative control after 1 h of toxin exposure. Furthermore, Zegura et al. [19] demonstrated that 0.5 µg/mL of CYN caused a significant up-regulation of the expression of BAX and BCL-2 genes (in favor of BCL2) in HPBLs, indicating the suppression of apoptosis after 24 h of exposure. These results suggest that the effects of CYN on the cell death mechanisms of immune cells depend on the different concentrations assayed and the varying susceptibility of cells to the toxin. However, higher proinflammatory cytokine production could explain the strong pro-apoptotic activity of CYN, as reported by Sieroslawska and Rymuszka [21]. In that sense, as reported in Section 2.3, the upregulation of TNF-α and INF-γ in Jurkat cells could be correlated with the high values of apoptotic cells found in this cell model.

The study of the molecular mechanisms of CYN toxicity is incomplete and primarily focused on the mRNA levels of genes involved in assessing cell and DNA damage [9]. To our knowledge, only a few studies have assessed the impact of CYN on the expression of genes implicated in pro-inflammatory and anti-inflammatory signaling [22,38]. For example, Bain et al. [38] found that in human liver cells and human dermal fibroblasts (HDF), CYN can up-regulate the gene expression of IL-6 and IL-8. Another study by Sieroslawska et al. [22] on fish immune cells demonstrated that CYN could increase the gene expression of pro-inflammatory (IL-1β and TNF-α) and, to a small extent, anti-inflammatory (transforming growth factor-β) cytokines. Nevertheless, the toxin did not affect IL-10 after 24 h of exposure. In relation to this, in the present work, we found high mRNA levels of IL-2 and INF-γ in both cell lines after exposure to the toxin. However, our results showed that THP-1 and Jurkat cell lines exposed to CYN had different inflammatory responses regarding gene expression (i.e., the upregulation of TNF-α in Jurkat but not in THP-1 after 24 h). This agrees with the study by Aceves et al. [39], who also reported a different pattern of intracellular pathways in THP-1 and Jurkat cells after exposure to chemotherapeutic drugs. Finally, it is worth mentioning that other works have studied the immune effects of other cyanotoxins. For example, Chen et al. [40] found that MC-LR could down-regulate the mRNA expression of TNF-α and INF-γ in murine macrophages. Furthermore, MC-LR increased the production of IL-6 but decreased the production of IL-2 in human lymphocytes [41]. It is important to highlight that despite the exposure of different immune cells to CYN, which could produce similar results concerning mRNA expression (i.e., upregulation of TNF-α or INF-γ), there are also contradictory results when compared with microcystins.

On the other hand, although Jurkat cells have high mRNA levels of TNF-α, the protein levels of this cytokine were not equivalent in the ELISA assay. Moreover, the levels of the other cytokines (IL-1β, IL-2, IL-6) were out of range. Therefore, we have not focused on whether the protein abundance of cytokines induced by CYN agreed with the transcriptional regulation. However, Hajighasemi and Mirshafiey [42] reported that in Jurkat cells, the production of some cytokines could be low. Therefore, considering it and comparing it to the gene expression results, Jurkat’s low release of cytokines could suggest that modifications (post-transcriptional/post-translational) of cytokine synthesis may occur. Furthermore, it is important to remember that the primary toxicity mechanism of CYN is the inhibition of protein synthesis, so gene expression and cytokine levels do not necessarily have to correlate between them.

Regarding THP-1 cells, CYN (2.56 µM) significantly increased the production of TNF-α after 4 h of incubation. However, the toxin did not cause an increased leakage of IL-2, IL-6 and IFN-γ. Similar results were obtained by Mosoova et al. [24] in murine macrophage RAW264.7 cells. They found that CYN induced the production of TNF-α, but it had no direct effect on the production of other pro-inflammatory mediators such as IL-6. Moreover, Adamovsky et al. [43] reported in the same cell line that another cyanotoxin (MC-LR) increased the production of TNF-α, but the levels of IL-6 were only slightly induced at the highest concentration of this toxin. These in vitro results agree with the in vivo results obtained by Diez-Quijada et al. [44]. They investigated the influence of oral CYN exposure (18.75; 37.5 and 75.0 µg CYN/kg b.w./day) on the mRNA expression and serum levels of different cytokines in the thymus and the spleen of male and female rats by RT-qPCR and ELISA, respectively. CYN produced immunomodulation mainly in the thymus, increasing the gene expression of IL-2, IL-6, TNF-α and IFN-γ. However, these values did not correlate with the cytokine levels measured in serum. In this sense, our results in vitro agree with those obtained in vivo.

Finally, THP-1 are monocytes that could differentiate into macrophages, thus participating in immune system responses. This differentiation process involves morphological and functional changes in monocytes [37,45]. Once differentiated, macrophages become long-lived cells and develop specialized functions. Among these important functions are the maintenance of tissue homeostasis and the response to microorganisms [46]. In addition, macrophages mediate innate immune responses and contribute to adaptive immunity. Considering this, knowing how CYN can affect this differentiation process is important. However, as far as we know, no works have studied the effects of CYN or other cyanotoxins in this process. In contrast, some studies have evaluated the effects of different types of toxins, such as mycotoxins [37,47], during the monocyte-to-macrophage differentiation process. In both studies, the toxin caused a decrease in the differentiation process after co-exposure with PMA. Similar results were found in our work, revealing that CYN reduced the monocyte differentiation at all the concentrations assayed after 48 h of exposure. These results show that toxins such as CYN could influence the decline of macrophage activation, which can compromise the immune system, affecting different physiological functions [48].

In summary, immune cell lines have a different sensitivity to CYN, affecting viability, monocyte differentiation, cell death mechanisms, cytokine levels, and gene expression.

## 4. Conclusions

CYN can exert an immunomodulatory effect on human immune THP-1 and Jurkat cells. This has been evidenced by changes in cell viability from 5 µM and 1 µM after 24 and 48 h of toxin exposure, respectively. The influence of CYN in the differentiation of THP-1 monocytes into macrophages was observed after 48 h of exposure. CYN also increased apoptosis and late apoptosis in Jurkat cells more severely than in THP-1. However, in this cell line, exposure for 48 h to 6 µM increased the percentage of necrosis. We observed more pro-inflammatory gene expression in both cell lines after 24 h of CYN exposure, with the highest up-regulation of IL-2 and IFN-γ. Moreover, in THP-1 cells, we found an increase in the IL-6 mRNA levels whereas, in Jurkat cells, the expression of TNF-α was up-regulated. However, the release of cytokines in the cell medium was not correlated with the mRNA levels as measured by qRT-PCR. Considering this, CYN could exert an immunomodulatory effect on human immune cells. Furthermore, additional studies (i.e., on the determination of other inflammatory markers such as inducible nitric oxide synthase or nitric oxide, the determination of reactive oxygen species levels, and the exploration of the activation of nuclear factor-κB) would be of interest to clarify the mechanisms involved in the immune system’s responses to this toxin.

## 5. Materials and Methods

### 5.1. Chemical and Reagents

CYN (95% purity) was purchased from Enzo Life Sciences (Lausen, Switzerland). Cell culture reagents were provided by Gibco (Biomol, Sevilla, Spain). Chemicals for cytotoxicity, differentiation and flow cytometry assays were obtained from Sigma-Aldrich (Madrid, Spain). Reagents for gene expression and cytokine detection were obtained from Bio-Rad Laboratories (Hercules, CA, USA) and Qiagen (Madrid, Spain).

### 5.2. Model System

Jurkat, a cell line derived from the peripheral blood of a patient with acute T-cell leukemia (ATCC^®^ TIB 152™), and THP-1, a cell line derived from peripheral blood from a patient with acute monocytic leukemia (ATCC^®^ TIB-202™), were maintained at 37 °C in an atmosphere containing 5% CO_2_ at 95% of relative humidity (CO_2_ incubator, Nuaire^®^, Murcia, Spain) at the Biology Service of the “Centro de Investigación, Tecnología e Innovación de la Universidad de Sevilla” (CITIUS). Cells were cultured in RPMI-1640 medium with high glucose (R8005) and supplemented with 10% heat-inactivated fetal bovine serum (FBS), 2 g/L sodium bicarbonate, 10,000 U/mL penicillin and 10 mg/mL streptomycin. Both cell lines were cultured following ATCC recommendations, and experiments were performed with culture passages 5–10.

### 5.3. Cytotoxicity and Differentiation

For the cytotoxicity tests, Jurkat and THP-1 cells were seeded in 96-well culture plates at a density of 5 × 105 and 3 × 105 cells/well, respectively. After 24 h, cells were exposed to different concentrations of CYN (0, 0.001, 0.01, 0.1, 1, 5 and 10 µM) for 24 and 48 h. The cytotoxicity of CYN was evaluated by MTS (3-(4,5-dimethylthiazol-2-yl)5-(3-carbox-ymethoxyphenyl)-2-(4-sulfophenyl)2H-tetrazolium salt) assay following the protocol described by Pichardo et al. [49].

To determine the effects of CYN on the differentiation of monocytic THP-1 cells into macrophages, cells were exposed to the toxin in the presence or absence of PMA (5 ng/mL) for 24 h and 48 h. Test concentrations were chosen considering their mean effective concentration (EC50) for 24 h (6.00 ± 1.04 µM) and 48 h (2.96 ± 0.67 µM), along with the fractions EC50/2 and EC50/4. The proportion of differentiated and nondifferentiated cells was determined according to Müller et al. [47]. Experiments were performed in triplicate.

### 5.4. Flow Cytometry

Jurkat and THP-1 cells were seeded at 1 × 10^6^ cells/well in 6-well tissue culture plates. Cells were exposed for 24 or 48 h to different CYN concentrations, chosen considering their EC50, 5.20 ± 1.20 and 6.00 ± 1.04 µM for Jurkat and THP-1, respectively, along with the fractions EC50/2 and EC50/4. Medium-treated cells were used as a negative control, and camptothecin (1.5 µM) as a positive control. Cells were centrifuged, washed with PBS, and resuspended in 500 µL of binding buffer. Cell suspensions (100 µL at 1 × 106 cells/mL) of each concentration assayed were incubated in 5 µL of Annexin V-FITC conjugate (marker for apoptosis) and 1 µL of Propidium Iodide (marker of necrosis) solution at room temperature for 15 min. After incubation, 400 µL of binding buffer was added, and the cell’s fluorescence was immediately determined by flow cytometry using a MACSQuant VYB (Miltenyi Biotec GmbH, Bergisch Gladbach, Germany) cytometer. Ten thousand events were collected per sample.

### 5.5. Gene Expression

Gene expression at the mRNA level was measured using the RT-qPCR. The gene expression of different cytokines, including IL-2, IL-6, IL-8, TNF-α and INF-γ, was assayed. GAPDH housekeeping gene was used as an internal control. Cells were exposed for 4 and 24 h to the 20% effective concentration (EC20) of CYN for Jurkat (2.14 ± 0.72 µM) and THP-1 (2.56 ± 0.63 µM) cells. Medium-treated cells were used as a negative control, and cells treated with a solution of 10 ng/mL LPS were used as positive control. After exposure, cells were centrifuged at 300× *g* for 6 min. RNA extraction was conducted using the RNeasy Mini Kit^™^ (Cat: 74104, Qiagen, Madrid, Spain) following the manufacturer’s instructions. An RNA purification step was performed using the RNase-free DNase set (Cat: 79254). The samples were stored at −80 °C until reverse transcription (RT). To obtain cDNA, RT was performed with 1 µg of total RNA using the QuantiTect^®^ reverse transcription kit (Cat: 205311, Qiagen, Madrid, Spain), as described by the manufacturer. Until RT-qPCR, the samples were kept at −20 °C. The cDNA was diluted (1:5) in RNase-free water and amplified in a LightCycler^®^480 system (Roche, Berlin, Germany) with the PrimePCR probe (Bio-Rad Laboratories, Inc., Hercules, CA, USA) for the corresponding cytokine: IL-2 (qHsaCIP0029918), IL-6 (qHsaCEP0051939), IL-8 (qHsaCEP0053894), TNF-α (qHsaCEP0040184), and INF-γ (qHsaCEP0050640); the iTaq Universal Probes Supermix (Cat: 1725134) was also included, in a final reaction volume of 10 µL (384-well plate). The protocol used for the amplification was 95 °C for 2 min followed by 50 cycles of 95 °C for 5 seg and 60 °C for 30 seg. The 2^−∆∆CT^ method was used to determine the results. Values above 1.5 were considered up-regulation, and values below 0.7 were considered down-regulation.

### 5.6. Cytokines Detection

The inflammatory response of Jurkat and THP-1 cells after 4 and 24 h of exposure to CYN (EC20) was analyzed with enzyme-linked immunosorbent assays (ELISA) for all cytokines studied (IL-2, IL-6, IL-8, TNF-α, IFN-γ). Medium-treated cells were used as a negative control, and LPS (10 ng/mL) as the positive control. The supernatants of the different samples were collected, and chemokine detection was conducted using Bio-Plex Pro Human Chemokine Assays (Cat. #171304090M), following the manufacturer’s instructions (Bio-Rad Laboratories, Inc., Hercules, CA, USA).

### 5.7. Statistical Analysis

The Kolmogorov–Smirnov test was used to confirm the normality of the distribution and the homogeneity of variances. Statistical analysis was carried out using the Kruskal–Wallis test, followed by Dunn’s multiple comparison tests for data that did not follow a normal distribution, and one-way ANOVA, followed by Tukey’s multiple comparisons test for data with a normal distribution. All analyses were performed with GraphPad Prisma (GraphPad Prism 9 Software Inc., La Jolla, CA, USA). Differences were considered significant at * *p* < 0.05, ** *p* < 0.01 and *** *p* < 0.001. EC50 values were derived by linear regression in the concentration–response curve. All experiments were performed at least three times.

## Figures and Tables

**Table 1:** Levels (pg/mL) of TNF-α measured in supernatant of THP-1 cells after 4 and 24 h of exposure to CYN effective concentration 20 (EC20) (2.56 µM). LPS (10 ng/mL) was used as positive control. Values are expressed as mean ± SD of two replicates. The significant levels observed are * *p* < 0.05, *** *p* < 0.001 compared to the negative control group.

THP-1	4 h	24 h
	Mean ± SD (pg/mL)	
	TNF-α	
C−	11.86 ± 0.38	35.72 ± 1.20
C+ (LPS 10 ng/mL)	139.67 ± 6.29 ***	554.39 ± 4.17 ***
CYN	17.45 ± 1.20 *	37.41 ± 2.75

## Data Availability

Not applicable.

## References

[1] Glibert P.M. (2020). Harmful algae at the complex nexus of eutrophication and climate change. Harmful Algae.

[2] Mehdizadeh Allaf M., Peerhossaini H. (2022). Cyanobacteria: Model Microorganisms and Beyond. Microorganisms.

[3] Díez-Quijada L., Prieto A.I., Guzmán-Guillén R., Jos A., Cameán A.M. (2019). Occurrence and toxicity of microcystin congeners other than MC-LR and MC-RR: A review. Food Chem. Toxicol..

[4] Dulić T., Svirčev Z., Malešević T.P., Faassen E.J., Savela H., Hao Q., Meriluoto J. (2022). Assessment of Common Cyanotoxins in Cyanobacteria of Biological Loess Crusts. Toxins.

[5] Weralupitiya C., Wanigatunge R.P., Gunawardana D., Vithanage M., Magana-Arachchi D. (2022). Cyanotoxins uptake and accumulation in crops: Phytotoxicity and implications on human health. Toxicon.

[6] Hercog K., Štampar M., Štern A., Filipič M., Žegura B. (2020). Application of advanced HepG2 3D cell model for studying genotoxic activity of cyanobacterial toxin cylindrospermopsin. Environ. Pollut..

[7] World Health Organization (2020). Cyanobacterial Toxins: Cylindrospermopsins. Background Document for Development of WHO Guidelines for Drinking-Water Quality and Guidelines for Safe Recreational Water Environments.

[8] Puerto M., Prieto A.I., Maisanaba S., Gutiérrez-Praena D., Mellado-García P., Jos A., Cameán A.M. (2018). Mutagenic and genotoxic potential of pure Cylindrospermopsin by a battery of in vitro test. Food Chem. Toxicol..

[9] Yang Y., Yu G., Chen Y., Jia N., Li R. (2021). Four decades of progress in cylindrospermopsin research: The ins and outs of a potent cyanotoxin. J. Hazard. Mater..

[10] Casas-Rodriguez A., Cameán A.M., Jos A. (2022). Potential Endocrine Disruption of Cyanobacterial Toxins, Microcystins and Cylindrospermopsin: A Review. Toxins.

[11] Wang L., Wang Q., Xiao G., Chen G., Han L., Hu T. (2020). Adverse effect of cylindrospermopsin on embryonic development in zebrafish (*Danio rerio*). Chemosphere.

[12] De la Cruz A.A., Chernoff N., Sinclair J.L., Hill D., Diggs D.L., Lynch A.T., Hiskia A.E., Triantis T.M., Antoniou M.G., Kaloudis T., Dionysiou D.D. (2020). Introduction to Cyanobacteria and Cyanotoxins. Water Treatment for Purification from Cyanobacteria and Cyanotoxins.

[13] Puerto M., Jos A., Pichardo S., Gutiérrez-Praena D., Cameán A.M. (2011). Acute effects of pure cylindrospermopsin on the activity and transcription of antioxidant enzymes in tilapia (*Oreochromis niloticus*) exposed by gavage. Ecotoxicology.

[14] Poniedziałek B., Rzymski P., Wiktorowicz K. (2012). First report of cylindrospermopsin effect on human peripheral blood lymphocytes proliferation in vitro. Cent. Eur. J. Immunol..

[15] Poniedziałek B., Rzymski P., Wiktorowicz K. (2014). Toxicity of cylindrospermopsin in human lymphocytes: Proliferation, viability and cell cycle studies. Toxicol. Vitr..

[16] Diez-Quijada L., Benítez-González M.D.M., Puerto M., Jos A., Cameán A.M. (2021). Immunotoxic Effects Induced by Microcystins and Cylindrospermopsin: A Review. Toxins.

[17] Poniedziałek B., Rzymski P., Kokociński M., Karczewski J. (2015). Toxic potencies of metabolite(s) of non-cylindrospermopsin producing *Cylindrospermopsis raciborskii* isolated from temperate zone in human white cells. Chemosphere.

[18] Poniedziałek B., Rzymski P., Karczewski J. (2015). The role of the enzymatic antioxidant system in cylindrospermopsin-induced toxicity in human lymphocytes. Toxicol. Vitr..

[19] Žegura B., Gajski G., Štraser A., Garaj-Vrhovac V. (2011). Cylindrospermopsin induced DNA damage and alteration in the expression of genes involved in the response to DNA damage, apoptosis and oxidative stress. Toxicon.

[20] Poniedziałek B., Rzymski P., Karczewski J. (2014). Cylindrospermopsin decreases the oxidative burst capacity of human neutrophils. Toxicon.

[21] Sieroslawska A., Rymuszka A. (2015). Effects of cylindrospermopsin on a common carp leucocyte cell line. J. Appl Toxicol..

[22] Sieroslawska A., Rymuszka A., Adaszek Ł. (2015). Effects of cylindrospermopsin on the phagocytic cells of the common carp (*Cyprinus carpio* L.). J. Appl Toxicol..

[23] Takser L., Benachour N., Husk B., Cabana H., Gris D. (2016). Cyanotoxins at low doses induce apoptosis and inflammatory effects in murine brain cells: Potential implications for neurodegenerative diseases. Toxicol. Rep..

[24] Moosova Z., Pekarova M., Sindlerova L.S., Vasicek O., Kubala L., Blaha L., Adamovsky O. (2019). Immunomodulatory effects of cyanobacterial toxin cylindrospermopsin on innate immune cells. Chemosphere.

[25] Ibrahim M.A.A., Goodman S.R. (2021). Cell Biology of the Immune System. Goodman’s Medical Cell Biology.

[26] Yasin Z.N., Mohd Idrus F.N., Hoe C.H., Yvonne-Tee G.B. (2022). Macrophage polarization in THP-1 cell line and primary monocytes: A systematic review. Differentiation.

[27] Chanput W., Mes J.J., Wichers H.J. (2014). THP-1 cell line: An in vitro cell model for immune modulation approach. Int. Immunopharmacol..

[28] Yunus M.A., Ramli M.M., Osman N.H., Mohamed R. (2021). Stimulation of Innate and Adaptive Immune Cells with Graphene Oxide and Reduced Graphene Oxide Affect Cancer Progression. Arch. Immunol. Ther. Exp..

[29] Abraham R.T., Weiss A. (2004). Jurkat T cells and development of the T-cell receptor signalling paradigm. Nat. Rev. Immunol..

[30] Chen J.L., Nong G.M. (2018). Advances in application of Jurkat cell model in research on infectious diseases. Zhongguo dang dai er ke za zhi = Chin. J. Contemp. Pediatr..

[31] Jarczak D., Nierhaus A. (2022). Cytokine Storm-Definition, Causes, and Implications. Int. J. Mol. Sci..

[32] Turner M.D., Nedjai B., Hurst T., Pennington D.J. (2014). Cytokines and chemokines: At the crossroads of cell signalling and inflammatory disease. Biochim. Biophys. Acta Mol. Cell Res..

[33] Sieroslawska A., Rymuszka A. (2014). Cylindrospermopsin induces oxidative stress and genotoxic effects in the fish CLC cell line. J. Appl. Toxicol..

[34] USEPA (2015). Health Effects Support Document for the Cyanobacterial Toxin Cylindrospermopsin.

[35] Schmeits P.C., Volger O.L., Zandvliet E.T., van Loveren H., Peijnenburg A.A., Hendriksen P.J. (2013). Assessment of the usefulness of the murine cytotoxic T cell line CTLL-2 for immunotoxicity screening by transcriptomics. Toxicol. Lett..

[36] Manyes L., Escrivá L., Ruiz M.J., Juan-García A. (2018). Beauvericin and enniatin B effects on a human lymphoblastoid Jurkat T-cell model. Food Chem. Toxicol..

[37] Solhaug A., Karlsøen L.M., Holme J.A., Kristoffersen A.B., Eriksen G.S. (2016). Immunomodulatory effects of individual and combined mycotoxins in the THP-1 cell line. Toxicol. Vitro.

[38] Bain P.A. (2007). Gene Expression Profiling of Cylindrospermopsin Toxicity. Ph.D. Thesis.

[39] Aceves M., Dueñas A., Gómez C., San Vicente E., Crespo M.S., García-Rodríguez C. (2004). A new pharmacological effect of salicylates: Inhibition of NFAT-dependent transcription. J. Immunol..

[40] Chen T., Zhao X., Liu Y., Shi Q., Hua Z., Shen P. (2004). Analysis of immunomodulating nitric oxide, iNOS and cytokines mRNA in mouse macrophages induced by microcystin-LR. Toxicology.

[41] Lankoff A., Carmichael W.W., Grasman K.A., Yuan M. (2004). The uptake kinetics and immunotoxic effects of microcystin-LR in human and chicken peripheral blood lymphocytes in vitro. Toxicology.

[42] Hajighasemi F., Mirshafiey A. (2016). In vitro Effects of Propranolol on T Helper Type 1 Cytokine Profile in Human Leukemic T Cells. Int. J. Hematol. Oncol. Stem Cell Res..

[43] Adamovsky O., Moosova Z., Pekarova M., Basu A., Babica P., Sindlerova L.S., Kubala L., Blaha L. (2015). Immunomodulatory potency of microcystin, an important water-polluting cyanobacterial toxin. Environ. Sci. Technol..

[44] Diez-Quijada L., Casas-Rodriguez A., Guzmán-Guillén R., Molina-Hernández V., Albaladejo R.G., Cameán A.M., Jos A. (2022). Immunomodulatory Effects of Pure Cylindrospermopsin in Rats Orally Exposed for 28 Days. Toxins.

[45] Daigneault M., Preston J.A., Marriott H.M., Whyte M.K., Dockrell D.H. (2010). The identification of markers of macrophage differentiation in PMA-stimulated THP-1 cells and monocyte-derived macrophages. PLoS ONE.

[46] Mosser D.M., Edwards J.P. (2008). Exploring the full spectrum of macrophage activation. Nat. Rev. Immunol..

[47] Müller G., Rosner H., Rohrmann B., Erler W., Geschwend G., Gräfe U., Burkert B., Möller U., Diller R., Sachse K. (2003). Effects of the mycotoxin ochratoxin A and some of its metabolites on the human cell line THP-1. Toxicology.

[48] Wang C., Ma C., Gong L., Guo Y., Fu K., Zhang Y., Zhou H., Li Y. (2021). Macrophage Polarization and Its Role in Liver Disease. Front. Immunol..

[49] Pichardo S., Jos A., Zurita J.L., Salguero M., Cameán A.M., Repetto G. (2007). Acute and subacute toxic effects produced by microcystin-YR on the fish cell lines RTG-2 and PLHC-1. Toxicol. Vitr..

